# A novel gene *SpCTP3* from the hyperaccumulator *Sedum plumbizincicola* redistributes cadmium and increases its accumulation in transgenic *Populus × canescens*

**DOI:** 10.3389/fpls.2023.1111789

**Published:** 2023-02-08

**Authors:** Shaocui Li, Renying Zhuo, Miao Yu, Xiaoyu Lin, Jing Xu, Wenmin Qiu, Haiying Li, Xiaojiao Han

**Affiliations:** ^1^ State Key Laboratory of Tree Genetics and Breeding, Key Laboratory of Tree Breeding of Zhejiang Province, Research Institute of Subtropical Forestry, Chinese Academy of Forestry, Hangzhou, Zhejiang, China; ^2^ Forestry Faculty, Nanjing Forestry University, Nanjing, Jiangsu, China; ^3^ College of Life Sciences, Fujian Agriculture and Forestry University, Fuzhou, China; ^4^ Institute of Virology and Biotechnology, Zhejiang Academy of Agricultural Sciences, Hangzhou, Zhejiang, China

**Keywords:** cadmium tolerance protein 3, *Sedum plumbizincicola*, cadmium stress, subcellular distribution, transgenic poplars

## Abstract

A cadmium (Cd) tolerance protein (*SpCTP3*) involved in the *Sedum plumbizincicola* response to Cd stress was identified. However, the mechanism underlying the Cd detoxification and accumulation mediated by *SpCTP3* in plants remains unclear. We compared wild-type (WT) and *SpCTP3*-overexpressing transgenic poplars in terms of Cd accumulation, physiological indices, and the expression profiles of transporter genes following with 100 μmol/L CdCl_2_. Compared with the WT, significantly more Cd accumulated in the above-ground and below-ground parts of the *SpCTP3*-overexpressing lines after 100 μmol/L CdCl_2_ treatment. The Cd flow rate was significantly higher in the transgenic roots than in the WT roots. The overexpression of *SpCTP3* resulted in the subcellular redistribution of Cd, with decreased and increased Cd proportions in the cell wall and the soluble fraction, respectively, in the roots and leaves. Additionally, the accumulation of Cd increased the reactive oxygen species (ROS) content. The activities of three antioxidant enzymes (peroxidase, catalase, and superoxide dismutase) increased significantly in response to Cd stress. The observed increase in the titratable acid content in the cytoplasm might lead to the enhanced chelation of Cd. The genes encoding several transporters related to Cd^2+^ transport and detoxification were expressed at higher levels in the transgenic poplars than in the WT plants. Our results suggest that overexpressing *SpCTP3* in transgenic poplar plants promotes Cd accumulation, modulates Cd distribution and ROS homeostasis, and decreases Cd toxicity *via* organic acids. In conclusion, genetically modifying plants to overexpress *SpCTP3* may be a viable strategy for improving the phytoremediation of Cd-polluted soil.

## 1 Introduction

Rapid economic and technological developments have resulted in the release of a relatively large amount of heavy metal waste, which has led to increased environmental pollution. In China, more than 1.3 × 10^5^ km^2^ of cultivated land has been polluted with cadmium (Cd) (MER and MLR, 2014; Jia et al., 2022). Soil degradation due to Cd contamination is becoming a major issue affecting food security and quality (Rehman et al., 2020). Heavy metal pollution has threatened the sustainable development of various industries (e.g., agriculture) (Yessica et al., 2020). Heavy metal pollution has attracted the attention of researchers in several disciplines (Li et al., 2018; Angulo-Bejarano et al., 2021). Cadmium is one of the most dangerous heavy metals because of its high toxicity and bioconcentration factor (Singh P et al., 2020; Cui et al., 2021) As a non-essential element in plants, Cd can disrupt plant metabolic activities, adversely affect photosynthesis, exacerbate the effects of nutrient deficiency as well as oxidative stress, and even inhibit cell proliferation and division (Rasafi et al., 2020; Shamelashvili et al., 2020; Yazdi, 2021). Cadmium stress can also inhibit plant growth in mild cases and cause plant death in severe cases (Yang et al., 2022). In heavily polluted areas, Cd can enter the human body through the food chain, with detrimental and irreversible effects on human health (Fu and Xi, 2020). Therefore, the remediation of Cd-contaminated soil is a major challenge that must be addressed (Wan et al., 2016; Han et al., 2020).

Phytoremediation has attracted widespread interest as an economically feasible and eco-friendly method for rescuing Cd-contaminated soils (Hamid et al., 2020; Raza et al., 2020). The Cd accumulation and detoxification capacities of plants are the main determinants of phytoremediation efficiency (Wang et al., 2021; Jia et al., 2022). The remediation of Cd-contaminated soil by hyperaccumulating plants is one of the most promising environmentally friendly remediation methods (Yaashikaa et al., 2022). It may be improved by increasing the absorption rate, transport, and accumulation of Cd in plants, which depends on the synergism between physiological and molecular mechanisms (Chai et al., 2020).

Elucidating the mechanisms regulating Cd tolerance and accumulation in plants is important for increasing the efficiency of the phytoremediation of Cd-polluted soil. Cadmium that is taken up by plants accumulates in the above-ground plant parts *via* the long-distance transport mediated by many transporters. Cd uptake, transport and distribution in plants depend on their metal transporters, such as zinc/iron-regulated transporter (ZRT/IRT-related ZRT-IRT-like proteins (ZIP)), heavy metal ATPase (HMA), Natural Resistance-Associated Macrophage Protein (NRAMP), and ATP-binding cassette (ABCC) (Zhang et al., 2013; Jiang et al., 2021; Yue et al., 2021). Cd in the external environment can be transported from the soil to the cell interior through the ZIP transporter on the plasma membrane (Viehweger, 2014). Then Cd^2+^ is transported to xylem through NRAMP, HMA, etc (He et al., 2015). The heterologous expression of *NRAMP3* from the hyperaccumulator *Sedum alfredii* in *Brassica juncea* reportedly does not significantly affect Cd tolerance, but it significantly increases the Cd content in the above-ground transgenic plant parts, implying that *SaNRAMP3* regulates the root-to-shoot translocation of Cd (Feng et al., 2018). *HMA* genes encode proteins that mediate the transport of metals from the roots to the shoots (Peng et al., 2017). In *Brassica rapa*, *HMA3* regulates Cd accumulation and increases the root-to-shoot Cd translocation rate (Zhang et al., 2019). In addition, some of the cadmium that enters the cytoplasm is transported into the vesicles by the action of ABC transporter and metal tolerance protein 1 (MTP1) (Lin and Aarts, 2012; Park et al., 2012).

The Cd tolerance of plants depends on physiological detoxification-related processes (Li et al., 2022). After passing through the cytoplasmic membrane, Cd is chelated with ligands to inhibit reactions between Cd and other cellular compounds. Plants often increase the accumulation of titratable acid to cope with heavy metal stress. When Cu was sprayed on leaves of *Hibiscus sabdariffa*, the contents of anthocyanin and titratable acid were increased significantly (Patricio et al., 2018). In lettuce, humic acid can stimulate production of organic acids by improving dark fixation of carbon dioxide or prevent the harmful effects of Cd, thereby increases titratable acidity of leaves (Haghighi et al., 2013). In response to lead stress, 5-aminolevulinic acid minimizes the potential for lead ions to inhibit metabolism by increasing the expression of genes encoding enzymes associated with malate and citrate metabolism, leading to the chelation of lead ions (Singh R et al., 2020).

Under heavy metal stress conditions, reactive oxygen species (ROS) levels increase substantially in plants. Heavy metal-tolerant plants then produce relatively large amounts of ROS-scavenging antioxidant enzymes to protect against heavy metal-induced oxidative stress (Dvorak et al., 2020). Superoxide dismutase (SOD) was inhibited under high Cd treatment, and excess ROS also led to significantly higher levels of oxidative damage (Zhang et al., 2020). A novel *Nicotiana tabacum* Cd transporter gene *NtNRAMP3* preventing chlorophyll degradation and reducing ROS accumulation under Cd stress conditions. Nevertheless, knockdown of the *NtNRAMP3* gene could increase the activities of SOD, POD and CAT thereby improving the Cd tolerance of tobacco to a great degree (Jia et al., 2022). Furthermore, *SaCu/Zn SOD* expression in *S. alfredii* is induced by Cd stress; the overexpression of this gene increases the resistance of transgenic *A. thaliana* plants to oxidative stress (Li et al., 2017).

New plant genes that may increase the efficiency of Cd phytoremediation were recently identified. For example, in *A. thaliana*, overexpressing the *IRON MAN* (*IMA*) gene encoding a small peptide improves Cd tolerance, whereas silencing *BRUTUS* (*BTS*) expression can increase the efficiency of the phytoremediation of Cd-contaminated soil (Zhu et al., 2020; Meng et al., 2022). Therefore, the functions of some key Cd stress-related genes in plants may be exploited to alleviate Cd toxicity and improve the Cd tolerance of plants. However, the physiological and molecular mechanisms underlying the functions of these novel genes related to Cd tolerance have not been precisely characterized, which is necessary for the sequential identification of novel genes. We previously detected five new proteins related to Cd tolerance or accumulation in the hyperaccumulator *Sedum plumbizincicola*, of which the production of Cadmium Tolerance Protein 3 (*SpCTP3*) is strongly induced by Cd treatments (Liu et al., 2019). Moreover, the expression of *SpCTP3*, which belongs to the *Nod19* superfamily, is up-regulated by stress. *Populus × canescens* (i.e., poplar species) is potentially useful for phytoremediations because of its fast growth rate and high biomass as well as the availability of an established genetic transformation system. The genetic transformation of this poplar species with Cd hyperaccumulation-related candidate genes from *S*. *plumbizincicola* may generate transgenic plants capable of accumulating high Cd levels, making them relevant for the phytoremediation of Cd-contaminated soil. Hence, the mechanisms controlling Cd accumulation, subcellular distribution, and chemical morphology in *SpCTP3-*overexpressing poplar trees under Cd stress conditions should be clarified. In this study, we examined the effects of *SpCTP3* on Cd absorption, distribution, and detoxification to improve its utility for Cd phytoremediation. The data presented herein will provide researchers with the theoretical basis for applying *SpCTP3* in the remediation of Cd-polluted lands by transgenic woody plants.

## 2 Materials and methods

### 2.1 Vector construction and plant transformation

The *S. plumbizincicola SpCTP3* gene was cloned by a PCR amplification using the primers SpCTP3-F/R. The full-length *SpCTP3* sequence comprises 1,383 base pairs. The amplified *SpCTP3* was incorporated into the pBI121 vector *via* homologous recombination for the subsequent expression under the control of the CaMV 35S promoter. An *Agrobacterium tumefaciens*-mediated leaf disc transformation method was used to insert *SpCTP3* into *P. × canescens* plants (Wen et al., 2022). Transgenic plants were identified by a genomic PCR amplification using the primers SpCTP3-F/R and the positive lines were further analyzed by quantitative real-time PCR (qRT-PCR). The relative expression levels were calculated according to the 2^−ΔΔCt^ method (Jia et al., 2022). The *TUBU* (Beta tubulin) gene was selected as an internal control. Primer details are listed in **Table S1**. Four-week-old transgenic rooted poplar plants and the control group were grown for 4 weeks in Hoagland nutrient solution (i.e., hydroponic system). The nutrient solution was renewed every 3 days. For the heavy metal treatment experiments, plants were grown for 30 days in Hoagland nutrient solution supplemented with 100 μmol/L CdCl_2_. The growth chamber conditions were set as follows: 16-h day (25°C)/8-h night (18°C), 50%–60% relative humidity, and a photon flux density of 120 μmol/m^2^/s. Plants were photographed and their phenotypes were analyzed.

### 2.2 Determination of Cd elements

The Cd-treated lines were harvested separately and rinsed thoroughly with tap water. They were then soaked in 20 mmol/L EDTA for 20 min and washed three times with deionized water. The roots, stems, and leaves of each line were collected and dried at 70°C until they reached a constant weight. At least 0.2 g dried material was used to measure the Cd concentration. Briefly, the samples were ground to fine powder, and digested with a mixture consisting of HNO_3_:HClO_4_ (9:1, v/v) at 120–200°C in a microwave-accelerated reaction system (CEM, Matthews, NC, USA). The final volume was adjusted to 25 mL with distilled water. The Cd concentration was analyzed using the iCAP-7400 inductively coupled plasma optical emission spectroscopy (ICP-OES) system (Thermo Fisher Scientific, California, USA). The heavy metal translocation factor was calculated according to the following formula (Dineshkumar et al., 2019): heavy metal content in the aerial plant parts/heavy metal content in the roots.

### 2.3 Separation of subcellular components and analysis of the Cd content

Fresh leaves and roots (2.0 g) were homogenized using a precooled mortar and 20 mL precooled extraction solution [50 mmol/L Tris-HCl, 250 mmol/L sucrose, and 1.0 mmol/L C_4_H_10_O_2_S_2_ (pH 7.5)]. After centrifuging the samples at 1,250 × g for 15 min, the precipitate was collected as the cell wall component. The supernatant was centrifuged at 20,800 × g for 45 min to obtain the precipitate (organelles) and supernatant (soluble fraction). All steps were completed at 4°C. After the collected components were digested, their Cd concentrations were determined by ICP-OES.

### 2.4 Extraction and quantitative analysis of Cd chemical forms

Cadmium in different chemical forms was extracted as described by Ouyang et al. (2019). Inorganic titratable acid cadmium (Cd_E_) was extracted with 80% ethanol, water-soluble cadmium (Cd_W_) was extracted with deionized water, Cd integrated with pectin and protein (Cd_NaCl_) was extracted with 1 mol/L NaCl, insoluble CdHPO_4_, Cd_3_(PO_4_)_2_, and other Cd-phosphate complexes (Cd_HAC_) were extracted with 2% HAC, oxalic acid bound cadmium (Cd_HCl_) was extracted with 0.6 mol/L HCl, and the rest was residual cadmium (Cdr). Approximately 0.5 g samples were shaken for the 22-h extraction at 25°C. The proportion of the sample weight and extraction solution volume was adjusted to 1:10 (w/v) before a centrifugation at 5,000 × g for 10 min. The precipitate was resuspended twice with the same extraction solution, shaken at 25°C for 2 h, and then centrifuged at 5,000 × g for 10 min. Finally, the supernatants after three centrifugations were combined. Each pooled solution was then evaporated on an electric plate to a constant weight. After digesting the materials, the Cd concentrations of the three parts were determined by ICP-OES.

### 2.5 Measurement of the Cd^2+^ net flux

To determine the net Cd^2+^ flow rate in different lines treated with Cd stress, nine fine roots (approximately 1.5 mm diameter) were selected from each Cd-treated transgenic line and the WT control. The Cd^2+^ net flux was measured according to the noninvasive micro-test (NMT) technique (NMT100 Series; Younger, USA LLC, Amherst, MA, USA) as previously described (He et al., 2015; Chen et al., 2017). Each fine root was equilibrated in the measurement solution (0.05mM CdSO_4_, 0.25 mmol/L NaCl, 0.1 mmol/L Na_2_SO_4_, 0.05 mmol/L KCl, 0.15 mmol/L MES, pH6.0) for 30 min and then transferred to fresh measurement solution before determining the Cd^2+^ net flux. The root Cd^2+^ flow rate was analyzed by moving the electrode between two points (30 μm) on the root surface to measure the voltage difference perpendicular to the root surface.

### 2.6 Analysis of antioxidants and antioxidative enzyme activities

To analyze antioxidant enzyme activities and the contents of ROS (O^2·−^ and H_2_O_2_), 1.0 g leaf and root samples were collected from the WT and transgenic plants and homogenized in 8 mL 50 mmol·L^−1^ sodium phosphate buffer (pH 7.8) using a prechilled mortar. The samples were then centrifuged at 10,000 × g for 15 min at 4°C. The supernatant was used to measure enzyme activities and the contents of ROS. The superoxide dismutase (SOD) activities were determined using NBT (Nitroblue tetrazolium) photoreduction method as absorbance 560 nm as described by Akbari et al. (2020). The peroxidase (POD) activity was assayed spectrophotometrically using hydrogen peroxide and guaiacol as extracts at 470 nm (Dai et al., 2020). Catalase (CAT) activity was tested in potassium phosphate buffer (pH 7.8) containing 3 mM H_2_O_2_ at 240 nm. The H_2_O_2_ content and the O2·^−^ level were determined as described by Alexieva et al. (2001) and Jing et al. (2020). H_2_O_2_ content was determined using trichloroacetic acid (0.1% w/v) at 630 nm (Alexieva et al., 2001). For determination of O^2·−^ level, supernatant was mixed with 10 mM of hydroxylamine hydrochloride and left for 20 min followed by the addition of sulfanilamide and naphthylamine. After 20 min of incubation at 25°C, absorbance was measured at 530 nm, and calculations were done using the standard curve of NaNO_2_ (Jing et al., 2020).

### 2.7 Determination of titratable acid contents

For titratable acid assay, 0.1 g sample of each treatment was added to water and then ground into a homogenate. The final volume was adjusted to 5 mL for the extraction of titratable acid. The titratable acid content was determined by a titration using sodium hydroxide (0.1 mol/L) and phenolphthalein (1%) as the pH indicator (Huo et al., 2009).

### 2.8 Analysis of transporter gene expression profiles

Total RNA was extracted from Cd-treated and WT poplar samples using the RNAprep Pure Plant Plus Kit (TIANGEN, Beijing), after which 800 ng total RNA in 20 μL was used to synthesize cDNA. The qRT-PCR analysis was performed using SYBR Green Premix Ex Taq II (TAKARA, Japan). The primer sequence refers to He et al. (2015) and Ma et al. (2014). Information regarding the qRT-PCR primers is provided in **Table S1**. The qRT-PCR reaction mixture was prepared in a volume of 20 μL, with 10 μL of SYBR green master mix, cDNA template (2 μL), ddH_2_O (6.8 μL), ROX Reference Dye 0.4μL and 0.4 μL of each of the primer. The qRT-PCR conditions were set as follows; 95°C for 30s, 40 cycles of 95°C for 5 s, and 60°C for 30 s, followed by melting of PCR products step: 95°C for 15 s, 60°C for 1 min, 95°C for 15 s. Each sample was analyzed using three technical replicates to ensure the results were reliable. Gene expression levels were calculated according to the 2^−ΔΔCt^ method, with *TUBU* selected as the reference gene. Relative gene expression levels were visualized in a heatmap on the basis of Z-score transformed values.

### 2.9 Statistical analysis

For each experiment, at least three plants were sampled. Statistical analyses were performed using the SPSS software (version 18.0). The significance of the differences between the treated and control samples was analyzed *via* a one-way ANOVA followed by Duncan’s test (*p*< 0.05). Data are presented herein as the mean ± standard deviation (SD). The Kruskal Wallis test was used if the data did not meet the assumptions for ANOVA (equality of variances) according to Levene’s test. A principal components analysis (PCA) was performed and the results were plotted using the ggbiplot package of R (version 3.6.4).

## 3 Results

### 3.1 Development of poplar lines overexpressing *SpCTP3*

Genomic DNA was extracted from the WT and transgenic lines using the CTAB method. The transgenic lines were verified using SpCTP3-F/R primers (**Table S1**). Of the 13 putatively transformed lines, 11 were confirmed as transgenic (**Figure S1**). The PCR results showed that *SpCTP3* was expressed in the transgenic poplar plants (**Figure S1**). Total RNA was extracted from some of the transgenic lines and reverse transcribed to cDNA for the qRT-PCR analysis. Three independent lines (OE-3, OE-6, and OE-7) with high *SpCTP3* expression levels were selected for the subsequent analyses.

### 3.2 *SpCTP3* has no effect on the biomass of the transgenic poplar under Cd treatment

**Figure 1:**
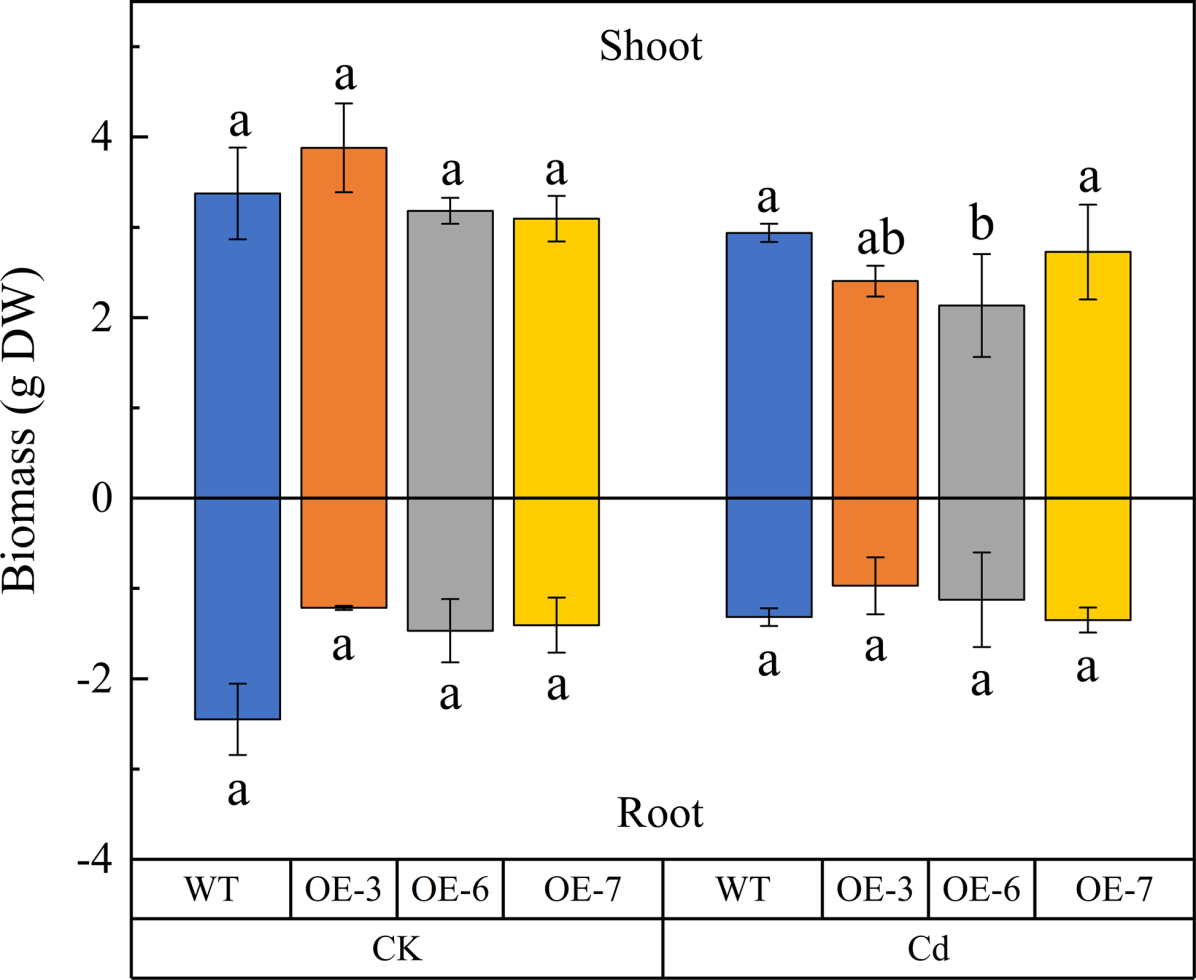
Effect of cadmium (Cd) stress on the shoot and root biomasses of *SpCTP3*-overexpressing transgenic and WT lines. Data are presented as the mean ± SD of three independent replicates. Different letters above bars indicate significant differences among treatments (*p*< 0.05; n = 3).

There was no significant difference in the dry weights of the untreated WT and transgenic plants. After the Cd treatment, the dry weight of the above-ground parts of the transgenic and WT plants decreased by an average of 28.49% and 12.96% (relative to the control), respectively. The dry weight of the root system decreased slightly after the Cd treatment, but this decrease was not significant. Accordingly, *SpCTP3* expression has no effect of the biomass of transgenic poplar plants exposed to Cd stress (**Figure 1**).

### 3.3 *SpCTP3* overexpression increased the Cd accumulation in transgenic poplars

**Figure 2:**
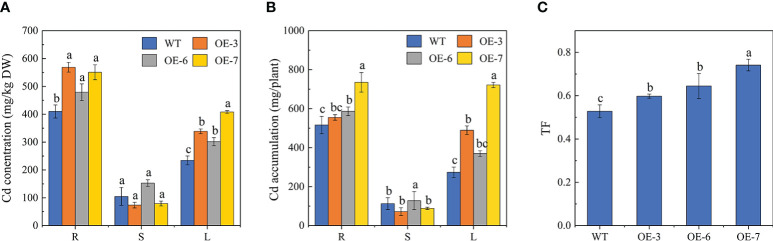
**(A)** Cd concentrations. **(B)** Cd accumulation. **(C)** Root-to-leaf Cd translocation factors (TFs).

To further functionally characterize *SpCTP3*, we measured the Cd content of specific parts of the WT and transgenic plants treated with Cd for 30 days (**Figure 2**). Notably, the root and leaf Cd contents were higher for the *SpCTP3*-overexpressing transgenic lines than for the WT controls. In contrast, there were no obvious differences in the stem Cd contents. The distribution of Cd was in the order of roots, leaves, and stems. The leaf Cd content was 48.58%–82.49% higher for the transgenic lines than for the WT controls. Similarly, the translocation factor was higher for the transgenic lines than for the WT plants. These observations indicated that compared with the WT plants, the transgenic poplar plants were better able to transport Cd from the roots to the aerial parts.

### 3.4 Overexpression of *SpCTP3* altered the distribution and chemical forms of Cd in transgenic plants

**Figure 3:**
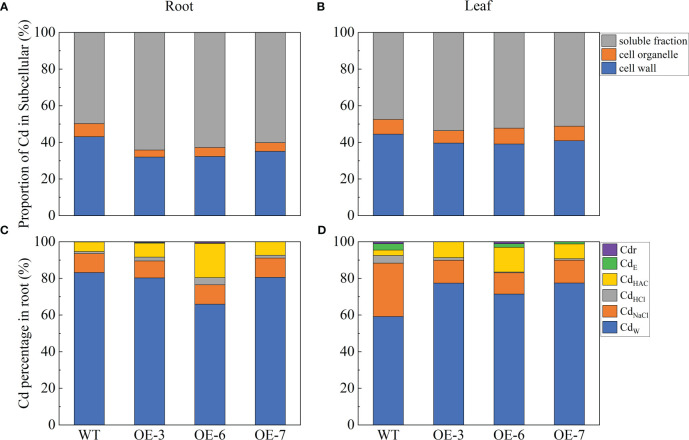
Cd subcellular distribution and Cd chemical forms in WT and *SpCTP3*-overexpressing poplar roots and leaves treated with 100 μmol·L^−1^ Cd for 30 days. **(A)** Cd subcellular distribution in the roots. **(B)** Cd subcellular distribution in the leaves. **(C)** Cd chemical forms in the roots. **(D)** Cd chemical forms in the leaves. Cd_E_, inorganic Cd; Cd_W_, water-soluble Cd; Cd_NaCl_, pectate- and protein-integrated Cd; Cd_HAc_, insoluble CdHPO_4_, Cd_3_(PO_4_)_2_, and other Cd-phosphate complexes; Cd_HCl_, oxalic acid-bound Cd; Cdr, Cd in residues.

In this study, Cd^2+^ was differentially distributed in various subcellular components. Most of the Cd in the roots and leaves of the transgenic lines was stored in the soluble fraction, followed by the cell wall and organelles (**Figure 3**). In the WT plants, 36.12% and 56.77% of the Cd were present in the cell wall and soluble fraction, respectively; the rest of the Cd was detected in the organelle fraction. In the WT leaves, 44.47% and 47.52% of the Cd content was distributed in the cell wall and soluble fraction, respectively. The cell wall Cd content was lower for the transgenic lines than for the WT plants, whereas the opposite pattern was observed for the Cd content in the soluble fraction.

Cadmium absorbed by plants is present in cells in diverse forms and affects plant growth and development. We extracted various forms of Cd and determined the Cd contents using an ICP-OES system. The Cd_W_ content was highest in the roots and leaves of the transgenic and WT plants (**Figure 3**). The Cd in the WT roots was mostly detected as Cd_W_ (59.19%), followed by Cd_NaCl_ (29.11%) and then Cd_HCl_, Cd_HAc_, and Cd_E_. The Cd in the transgenic roots was also mostly detected as Cd_W_ (average of 75.59%), although the Cd_HAc_ content was also relatively high. The Cd in the leaves of the transgenic and WT plants was predominantly in the Cd_E_, Cd_NaCl_, and Cd_HAc_ forms. Compared with the WT control, the Cd treatment of the transgenic plants decreased the Cd_W_ and Cd_NaCl_ contents, while increasing the Cd_HCl_ and Cd_HAc_ contents. Hence, the overexpression of *SpCTP3* altered the Cd distribution and chemical forms in the transgenic plants.

### 3.5 Overexpression of *SpCTP3* enhanced the net flux of Cd^2+^ in poplar roots

**Figure 4:**
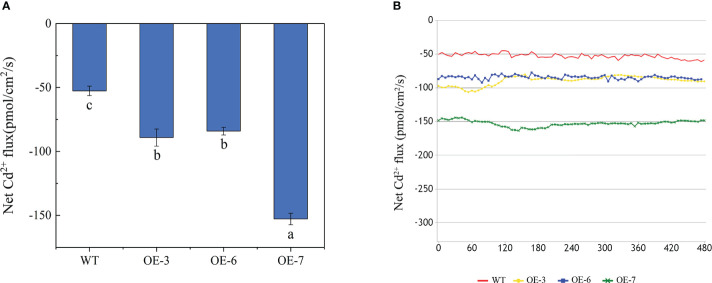
Effects of Cd treatments on the Cd^2+^ net flux in the roots of WT and transgenic lines. **(A)** Results of the statistical analysis of the steady-state Cd^2+^ net flux. **(B)** Time-course of the Cd^2+^ net flux at the root surface. Different letters on bars indicate significant differences in the Cd concentrations among lines (*p*< 0.05).

To clarify the changes in Cd uptake by the roots of transgenic and WT poplar plants exposed to Cd stress, we used the NMT technique to analyze the Cd^2+^ flow rate at 120 μm from the root tip after the Cd treatment. The Cd^2+^ flow rate was 72.927–163.48 pmol/cm^2^/s for the transgenic lines and 44.707–61.058 pmol/cm^2^/s for the WT plants (**Figure 4**). The net influx of Cd^2+^ was higher for the transgenic roots than for the WT roots.

### 3.6 Overexpression of *SpCTP3* increased the ROS content and affected antioxidant enzyme activities

**Figure 5:**
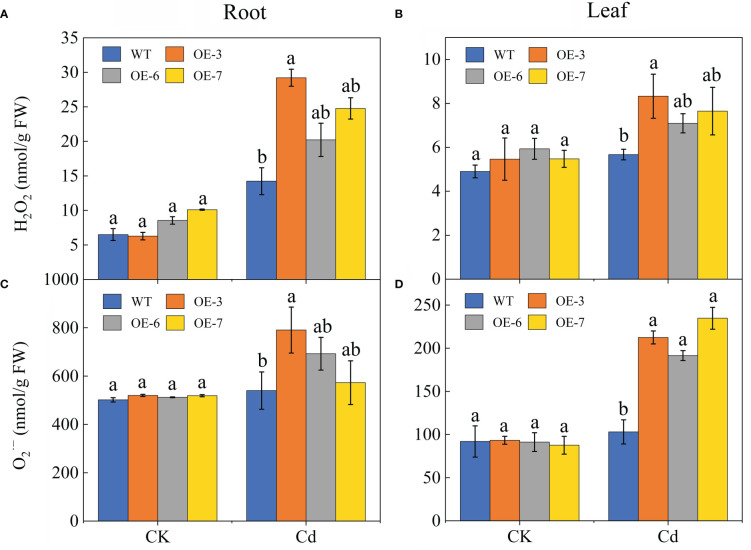
Effect of Cd stress on the ROS contents of the WT and *SpCTP3*-overexpressing transgenic poplar roots and leaves. **(A)** Root H_2_O_2_ concentrations. **(B)** Leaf H_2_O_2_ concentrations. **(C)** Root ${\text{O}}_{2}^{\cdot -}$ concentrations. **(D)** Leaf ${\text{O}}_{2}^{\cdot -}$ concentrations. Data are presented as the mean ± SD of three independent replicates. Different letters above bars indicate significant differences among treatments (*p*< 0.05).

An exposure to Cd stress results in different degrees of oxidative stress in plants. The ${\text{O}}_{2}^{\cdot -}$ and H_2_O_2_ concentrations were compared between the WT and transgenic poplar plants (**Figure 5**). For both the WT and transgenic samples, the Cd treatment induced a significant H_2_O_2_ and ${\text{O}}_{2}^{\cdot -}$ burst. The leaf ${\text{O}}_{2}^{\cdot -}$ content was 2-times higher in the transgenic plants than in the WT plants. In the roots, the H_2_O_2_ and ${\text{O}}_{2}^{\cdot -}$ contents increased in the transgenic plants by varying degrees, resulting in levels that were significantly higher than those in the WT plants. Additionally, H_2_O_2_ and ${\text{O}}_{2}^{\cdot -}$ accumulated more in the leaves than in the roots, although this phenomenon was significantly less pronounced in the WT plants than in the transgenic plants.

**Figure 6:**
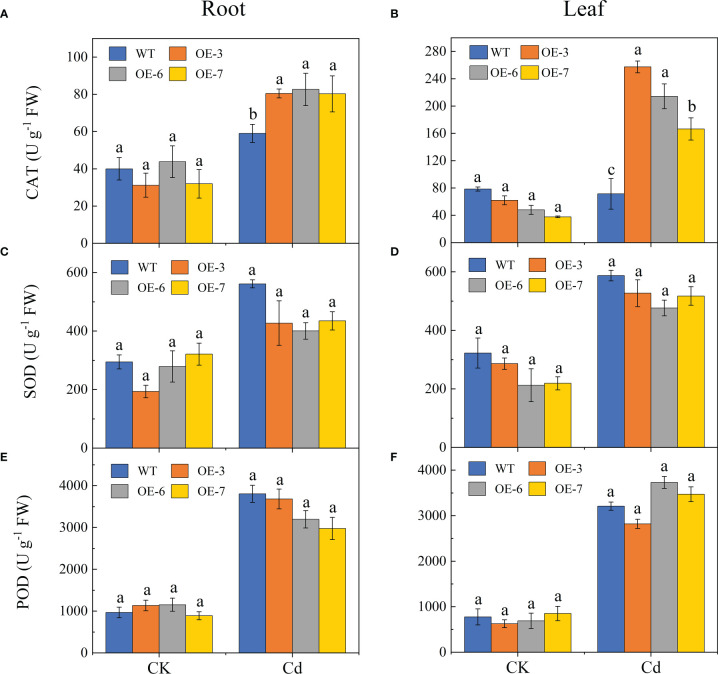
Effect of Cd stress on the antioxidant enzyme activities of the WT and *SpCTP3*-overexpressing poplar roots and leaves. **(A)** Root CAT activities. **(B)** Leaf CAT activities. **(C)** Root SOD activities. **(D)** Leaf SOD activities. **(E)** Root POD activities. **(F)** Leaf POD activities. Data are presented as the mean ± SD of three independent replicates. Different letters above bars indicate significant differences among treatments (*p*< 0.05).

Cadmium significantly affected the CAT, SOD, and POD activities (**Figure 6**). The CAT activity levels were significantly higher in the transgenic lines than in the WT plants after the Cd treatment. Compared with the WT control, the CAT activity was respectively 1.37-fold and 2.98-fold higher in the roots and leaves of the transgenic lines by Cd stress. Furthermore, there was no significant difference in the SOD activities of the Cd-treated transgenic and WT leaves, but the root SOD activities were slightly lower in the transgenic lines than in the WT control following the Cd treatment. In addition, the POD activity in the leaves was slightly higher in the transgenic lines than in the WT plants, but there was no significant difference in the root POD activity.

### 3.7 Overexpression of *SpCTP3* increased the titratable acid content of Cd-treated transgenic lines

**Figure 7:**
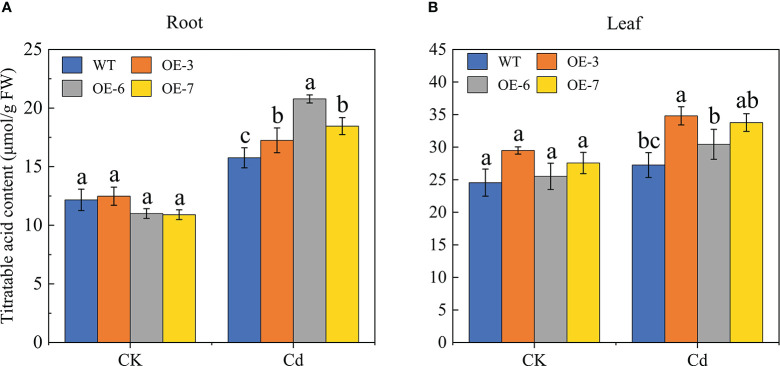
Titratable acid contents of the WT and *SpCTP3*-overexpressing transgenic poplar roots and leaves treated with 100 μmol·L^−1^ Cd for 30 days. **(A)** Root titratable acid contents. **(B)** Leaf titratable acid contents. Data are presented as the mean ± SD of three independent replicates. Different letters above bars indicate significant differences among treatments (*p*< 0.05).

Titratable acids are often considered to be important for the resistance of plants to heavy metal pollution because they contribute to the chelation of metal elements and convert metal ions to less toxic or non-toxic chelated states. Titratable acids were extracted from the *SpCTP3*-overexpressing and WT plants and the total organic acid content was determined *via* acid-base titration (**Figure 7**). The Cd treatment induced a substantial change in the titratable acid content. More specifically, the titratable acid content in the transgenic roots and leaves increased significantly, peaking at 20.76 μmol·g^−1^ in the roots (i.e., 31.87% higher than the titratable acid content of the control roots) and 30.81 μmol·g^−1^ in the leaves. Thus, the overexpression of *SpCTP3* increased the titratable acid content of transgenic lines under Cd stress conditions.

### 3.8 Expression of the genes involved in Cd^2+^ transport and detoxification

**Figure 8:**
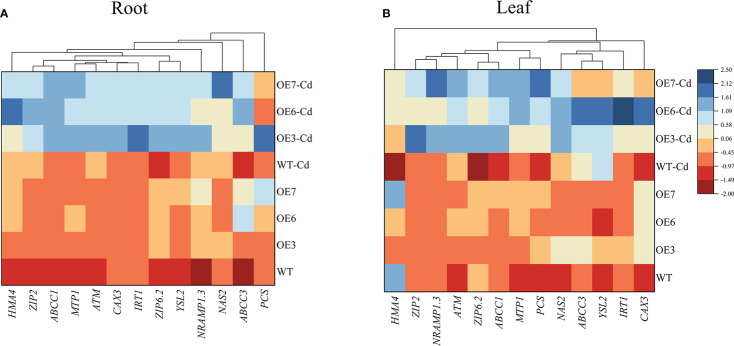
Expression levels of key transporter genes in the Cd-treated roots and leaves of WT and transgenic poplar lines treated with 0 or 100 μmol·L^−1^ Cd^2+^ for 30 days. **(A)** Root gene expression levels. **(B)** Leaf gene expression levels. Three biological replicates were analyzed. Red and blue respectively indicate negative and positive Z-scores for the gene expression levels.

On the basis of the physiological data (**Figure 8**), differences in the Cd uptake by the roots and the subsequent translocation to the shoots may be a possible reason for diversity in the Cd accumulation between the WT and transgenic poplar plants. Therefore, we focused on the transcriptional regulation of some transporter genes involved in Cd^2+^ transport and detoxification. The expression levels of all 12 genes related to Cd transport and detoxification were differentially up-regulated in the roots after the Cd treatment (**Figure 8**). In the roots, the up-regulated expression of *ZIP2* (encoding zinc-regulated transporter 2), *ZIP6.2*, and *IRT* (encoding an iron-regulated transporter-related protein) was greater in the transgenic lines than in the WT control. Among the leaves, the most up-regulated genes were *IRT*, *YSL2* (encoding a yellow stripe-like protein), and *CAX* (encoding a cation exchanger). Moreover, the Cd treatment significantly up-regulated the expression of *HMA4* (encoding heavy metal ATPase 4) only in the transgenic roots. Similar expression trends were observed for other genes in the leaves and roots, in which *NAS* (encoding a nicotianamine synthase), *MTP1* (encoding metal tolerance protein 1), and *Nramp1.3* (encoding natural resistance-associated macrophage protein 1.3) expression levels increased significantly in the Cd-treated transgenic lines.

### 3.9 Principal component analysis of physiological responses

**Figure 9:**
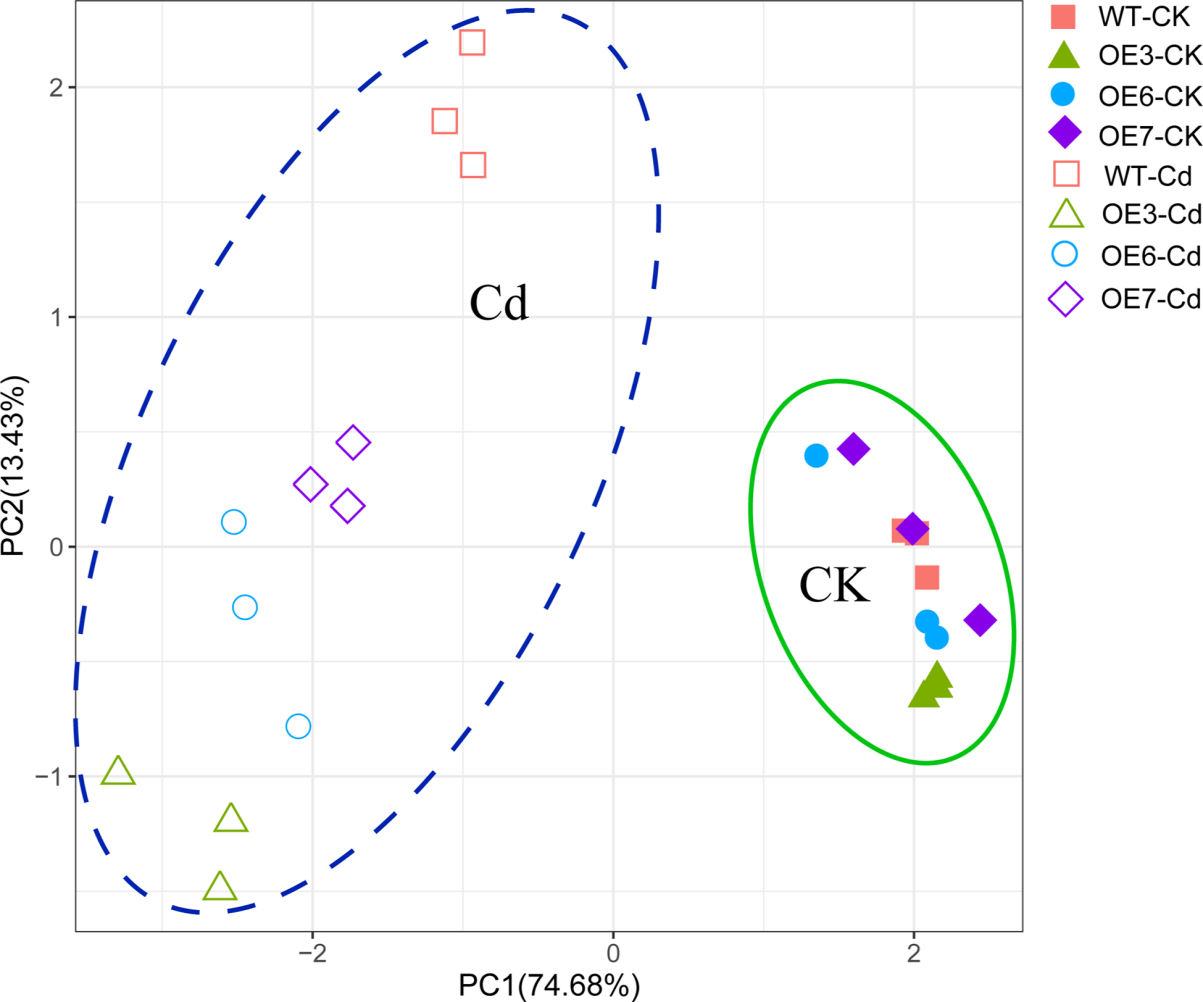
Principal component analysis plots of the Cd concentrations and ROS, antioxidant, and titratable acid contents in the roots and leaves of WT and transgenic plants.

To determine the response patterns of the WT and transgenic poplar plants to Cd^2+^, a PCA was conducted using the physiological data related to the exposure to Cd^2+^ (e.g., Cd concentration as well as ROS, antioxidant, and titratable acid contents) (**Figure 9**). The PCA results indicated that 88.11% of the total variance could be explained by the effects of the Cd stress on the WT and *SpCTP3*-overexpressing transgenic poplar plants, with principal components 1 and 2 respectively explaining 74.68% and 13.43% of the Cd-induced variance. There was a significant difference between the *SpCTP3*-overexpressing transgenic and WT poplar plants under Cd stress conditions.

## 4 Discussion

### 4.1 *SpCTP3* overexpression enhanced Cd accumulation by regulating subcellular components and Cd chemical forms

The soluble fraction of cells consists mainly of vesicles, which contain many proteins, sugars, and organic acids that bind to heavy metals to decrease their effects and limit the amount of heavy metals entering organelles, thereby protecting cells from Cd stress (Li et al., 2014; Ouyang et al., 2019). For example, in *Capsicum annuum* roots, the Cd content in the soluble fraction can be as high as 77%–87% (Xin and Huang, 2014). Additionally, Cd is mainly distributed in the soluble fraction of the leaves and roots of Cd-tolerant radish varieties (Xin et al., 2017). We obtained similar results. In the WT control, the soluble fraction had the highest Cd content (56.77%), followed by the cell wall. In the transgenic lines, the rank-order for the Cd content was as follows: soluble fraction > cell wall > organelle (**Figure 3**). This trend was also detected in the leaves. Plants absorb Cd and store most of it in the soluble fraction, followed by the cell wall. However, plant responses to Cd stress may vary among species and stress levels. The transgenic poplar plants had a higher Cd capacity than the WT controls. The accumulation of Cd in the cell wall and soluble fraction can effectively decrease Cd toxicity in plants. In the current study, the Cd content decreased in the cell walls of the transgenic roots and leaves, whereas it increased in the soluble fraction. Hence, in the *SpCTP3*-overexpressing transgenic poplar plants, Cd ions were separated and fixed in the cytoplasm in the form of relatively stable metal chelates/complexes. Compared with the WT plants, the overexpression of *SpCTP3* in the transgenic plants increased the Cd content of the roots and leaves, while also altering the subcellular distribution of Cd by increasing and decreasing the Cd proportions in the soluble fraction and cell wall, respectively. These findings reflect the importance of *SpCTP3* for minimizing the toxic effects of Cd in plants.

Under heavy metal stress conditions, multiple mechanisms may be activated in plants to prevent the excessive accumulation of metals in the cytosol and limit the toxicity of the metals. If heavy metals enter the soluble part of the cell, the internal heavy metal tolerance mechanism is activated, resulting in the production of metal chelators, including organic acids, amino acids, and binding proteins (Hall, 2002). Organic acids are an extremely important class of metal ligands that are crucial for the absorption, transport, storage, and detoxification of heavy metals (Long et al., 2003). The proportion of the Cd content in the cell wall of the roots and leaves of the transgenic poplar plants decreased, which was in contrast to the increase in the proportion of the Cd content in the soluble fraction, implying that more Cd ions in cytoplasm need to be immobilized in the form of stable metal chelates/complexes in the transgenic poplar plants than in the WT plants. Accordingly, poplar plants can avoid the harmful effects of Cd stress by decreasing the free Cd concentration in the cytoplasm. In response to the 30-day Cd treatment in this study, the titratable acid content was significantly higher in the transgenic poplar plants than in the WT plants. This indicates that *SpCTP3* is critical for ameliorating the toxicity of Cd in plants.

The chemical form of Cd reflects the degree of migration and the toxicity of Cd (Kudo et al., 2021). Highly mobile inorganic and organic Cd compounds are usually more toxic than Cd_HAc_, Cd_HCl_, and Cd_NaCl_ (Wu et al., 2016). In our study, Cd_W_ was the dominant form in all transgenic lines. Additionally, the Cd_W_ content was lower in the roots of the transgenic lines than in the WT roots. The Cd_W_ form of Cd is converted to Cd_HAc_ and Cd_HCl_. However, Cd_HAc_ is associated with Cd uptake, transport, and detoxification (Qiu et al., 2011). It is also indicative of the adaptive response of plants to an exposure to stress. Cd_W_ extracted as free inorganic Cd, and these free Cd^2+^ are not only easily localized in the organelles, but it is also transported to the above-ground tissues. Our analysis of leaves showed that Cd_W_ was more abundant in the transgenic lines than in the WT plants, indicating that the transgenic lines had considerably more free Cd^2+^ (Xin et al., 2017). The Cd content as well as the translocation factor were higher in the transgenic lines than in the WT plants, which is consistent with the results of a recent study (Lai et al., 2021). In the WT control, the proportion of Cd_NaCl_ increased, indicative of the increase in the binding between Cd and pectin or protein. Therefore, the leaf cell wall Cd content was higher for the WT plants than for the transgenic lines. Both Cd_HAc_ and Cd_HCl_ are mainly present in the cell wall and vesicles (Jiang et al., 2007). In the current study, Cd_HAc_ and Cd_HCl_ contents were higher in the transgenic lines than in the WT plants, which corresponds to the subcellular distribution of Cd. In summary, the conversion of Cd to Cd_HAc_, Cd_HCl_, and Cd_NaCl_ is an important mechanism related to the Cd uptake, translocation, and detoxification in plants.

### 4.2 *SpCTP3* induced oxidation reaction under cadmium treatment

Plants take up heavy metals from the soil primarily through their roots. Therefore, determining the Cd uptake and efflux from the roots is important for measuring plant Cd accumulation. Noninvasive micro-test techniques can measure real-time ion transport changes in living plant and animal tissues, cells, and internal/external environments. Our NMT analysis revealed that the Cd in-flow rate was significantly higher for the transgenic roots than for the WT roots. The overexpression of *SpCTP3* promoted the influx of Cd in root cells and the accumulation of Cd, indicating that the roots of the transgenic lines had a greater Cd uptake capacity than the WT roots, which is consistent with the findings of an earlier study by Zhang et al. (2021).

In our study, *SpCTP3* was induced to be expressed by the Cd treatment and Cd accumulation was increased in the transgenic lines. Then ROS levels were significantly elevated, which induced the up-regulated expression of *SpCTP3*, thereby promoting ROS detoxification. This is one of the reasons for the increased antioxidant enzyme activities in the *SpCTP3*-overexpressing transgenic lines. In addition, Cd induced the accumulation of ROS in the roots and leaves, ultimately resulting in oxidative stress, which is in accordance with the findings of recent studies involving other plant materials (Bari et al., 2019; Zhang et al., 2020a). In the current study, Cd stress induced the excessive accumulation of ${\text{O}}_{2}^{\cdot -}$ in the roots and leaves. Moreover, the H_2_O_2_ content increased as the treatment duration increased. To protect against excessive ROS, plants employ a complex array of antioxidant defense mechanisms to prevent or mitigate the oxidative damage caused by abiotic stresses (Shafi et al., 2022). Because they are signaling compounds, the accumulation of H_2_O_2_ and ${\text{O}}_{2}^{\cdot -}$ may increase the activities of CAT, SOD, POD, and other enzymes during plant responses to Cd stress (Wrzaczek et al., 2013). Superoxide dismutase activity is positively correlated with the intracellular ROS level within a certain range (Zhou et al., 2021). Under stress conditions, an increase in the ROS content increases the activities of SOD and other antioxidant enzymes (Gupta et al., 2017). In addition, SOD converts superoxide radicals into less toxic substances in reactions that produce H_2_O_2_ (Fujita and Hasanuzzaman, 2022). This may explain the observed accumulation of H_2_O_2_ in the transgenic poplar lines. Subsequently, CAT and POD are activated as H_2_O_2_ scavengers to maintain plant redox homeostasis (Tadjouri et al., 2022). In our study, the *SpCTP3*-overexpressing transgenic lines had significantly elevated CAT and POD activities after the Cd treatment, and the CAT activities were significantly higher in the Cd-treated transgenic lines than in the WT plants (**Figure 6**). Similarly, Cd stress reportedly causes the SOD, POD, and CAT activities in the above-ground parts of rice plants to increase to some extent to limit the toxic effects of Cd (Afzal et al., 2018). An exposure to Cd stress can significantly increase SOD, CAT, APX, and POD activities in marigold (*Calendula calypso*) by 150%, 79%, 97%, and 60%, respectively, leading to the conversion of H_2_O_2_ to non-toxic oxygen and water (Farooq et al., 2020).

### 4.3 *SpCTP3* overexpression enhanced Cd transport by up-regulating the expression of transporter genes

The physiological process leading to the accumulation of Cd in above-ground plant parts is divided into the following three stages: root cell absorption; ectoplastic pathway-, symplast pathway-, and apoplast pathway-mediated transport; and above-ground accumulation (Uraguchi et al., 2009). Cadmium is transported to the above-ground tissues *via* numerous transporter proteins. In plants, the NRAMP proteins form one of the heavy metal transporter families in monocotyledons and dicotyledons (Ishida and Corcino, 2022). Their functions have been demonstrated in rice (Chang et al., 2020), poplar (Chen et al., 2019), and other plants (Li et al., 2021).

The ability of roots to absorb Cd is the decisive factor influencing the accumulation of Cd in plant shoots (Chaney, 2015). The overexpression of the *S. alfredii SaNRAMP1H* gene significantly increases the Cd contents of tobacco plants (above-ground and below-ground parts), implying that *SaNRAMP1H* promotes Cd uptake and accumulation (Zhang et al., 2020). The ZIP family proteins also function as heavy metal transporters. A previous study showed that Cd^2+^ can enter plant root cells through the ZIP transporter, which regulates Cd uptake, sequestration, and translocation (Ma et al., 2014). In addition, the overexpression of the *Vicia sativa VsIRT1* gene in *A. thaliana* enhances the transport and accumulation of Cd (Zhang et al., 2020b). In poplar, *NRAMP1.3*, *ZIP2*, and *ZIP6.2* encode plasma membrane proteins that control the entry of Cd^2+^ into root cells. In the current study, the Cd treatment up-regulated the *NRAMP1*, *ZIP2*, and *ZIP6.2* expression levels and increased the cytoplasmic Cd content in the transgenic lines; the Cd content was higher in the transgenic lines than in the WT plants (**Figure 8**).

Cytosolic Cd^2+^ may bind to phytochelatins (PCs), which are synthesized from glutathione in a reaction catalyzed by PC synthase (PCS) (Liu et al., 2021). The generated PC–Cd complex can be transported to vesicles *via* ABC transporters (e.g., ABCC1 and ABCC2) (Feng et al., 2020). Cytoplasmic Cd^2+^ may also be transported into vesicles *via MTP1*, which is located in the vesicle membrane (Singh et al., 2021). In poplar, *PCS*, *ABCC1*, and *MTP1* are differentially expressed in response to Cd^2+^. In the present study, *PCS*, *ABCC1*, and *MTP1* expression levels increased 3.7-fold, 7.4-fold, and 16.4-fold, respectively, in the Cd-treated transgenic poplar plants. Earlier research confirmed *HMA4* is localized to the plasma membrane, wherein it helps export divalent cations (e.g., Cd^2+^/Zn^2+^) from the cytoplasm and facilitates their transport into the xylem (He et al., 2015; Wang et al., 2020). A recent study indicated *HMA4* is responsible for the transport of Cd^2+^ through the central cylinder and xylem (Wiyono et al., 2021). In the root system, *HMA4* expression increased significantly in the transgenic lines exposed to Cd stress, which increased the transport of Cd from the roots to the above-ground parts. Moreover, *HMA4* expression was up-regulated in the roots, but not in the leaves. These results suggest that the substantial accumulation of Cd in the transgenic lines may have been due to enhanced heavy metal transport.

## 5 Conclusion

Transgenic poplar plants overexpressing *SpCTP3* accumulated more Cd in the above-ground tissues than the WT plants, likely because of changes to Cd distribution and chemical forms. The transgenic poplar lines also had a higher net influx of Cd^2+^ into the roots than the WT controls. Consistent with this observation, the expression levels of transporter genes (e.g., *ABCC1*, *MTP1*, and *ZIP6.2*) involved in Cd^2+^ transport and detoxification, were higher in the transgenic roots and leaves than in the WT roots and leaves after the Cd^2+^ treatment. Moreover, in response to the exposure to Cd stress, the ${\text{O}}_{2}^{\cdot -}$ , H_2_O_2_, and titratable acid concentrations in the roots and leaves were higher for the transgenic lines than for the WT plants. These findings indicate that the overexpression of *SpCTP3* promoted the accumulation of Cd by increasing the organic acid content, up-regulating the expression of key transporter genes, and regulating oxidative homeostasis. The results of this study provide a theoretical basis for improving plant characteristics *via SpCTP3* expression to increase the utility of poplar plants for remediating Cd-contaminated soil.

## Data availability statement

The original contributions presented in the study are included in the article/**Supplementary Material**. Further inquiries can be directed to the corresponding authors.

## Author contributions

XH, SL and RZ designed the experiments. SL performed the experiments and conducted the data. SL and XH wrote the manuscript. XH contributed to manuscript revision. MY, XL, WQ, JX and HL analyzed the data. All authors contributed to the article and approved the submitted version.
