# Supplementary material for: A novel gene SpCTP3 from the hyperaccumulator Sedum plumbizincicola redistributes cadmium and increases its accumulation in transgenic Populus × canescens
Source: Front Plant Sci. 2023 Feb 8;14:1111789. doi: 10.3389/fpls.2023.1111789 (PMC9945123; doi:10.3389/fpls.2023.1111789)
Supplement: Supplementary file 1 [file DataSheet_1.docx]

| Gene name | Primer-Forward | Primer-Reverse | Description |
| --- | --- | --- | --- |
| *SpCTP3* | ATGGTGAGTAGGGGACATTTTGC | TTACATTACAATGGACTGGTAACCAT | Primers used in gene detection |
| q*SpCTP3* | TTTGATGCGGAGGTTGTGGA | TCGCTGAACCTCTAAGTCCC | Primers used in qPCR detection |
| *YSL* | GCTGCCCTCTTTGTGCTA | TTGCGGTGCCAATAACTT | Yellow stripe-like protein |
| *NAS* | TCCTGGTGCTATATTGATGC | GGCGAACACATGGATAGAG | Nicotianamine synthase |
| *ABCC1* | CAAGTGGCACGATGAGTCT | AGGGACGGAACTCTACAGATG | ATP-binding cassette transporter |
| *ABCC3* | GATTTCCCCACTAGCCAAA | TGAAGCAGTAAAGCCACGTA | ATP-binding cassette transporter |
| *CAX3* | GCACACAACATGTCATCCTC | AAAGCCATAGCATTGAGCAG | Cation exchanger |
| *PCS1* | TTACTTCCAGACACAGTCGGA | GAGCCAAACACACTAGCTTC | Phytochelatin synthetase |
| *IRT1* | GCTGCTCTTTGCTTCCAT | TAGGAGATCAACCAATGCC | Zinc/iron regulated transporter related protein |
| *ZIP2* | TGCCATCAACCATCTCATAG | ACAAAGGAAAGATGAACTCCC | Zinc-regulated transporters and iron-regulated transporter-like protein |
| *ZIP6.2* | ACGACGACAGTAACCCCAAT | TAGCCTTCAGAAACAAGAGCAC | Zinc-regulated transporters and iron-regulated transporter-like protein |
| *Nramp1.3* | TGAAAATCCGCAAATCGC | TCCACAAAGATGTTCGGTCAT | Natural resistance associated macrophage protein |
| *MTP1* | AGATTGAAAAGGGTCTGC | CTCTCAGAAACATCACGC | Metal tolerance protein |
| *HMA4* | AAGTATTCTTGCCTGCTC | CCTTAACAATTTGGAGCT | P-type heavy metal ATPase |
| *TUBU* | AAGGATGCCTATGTTGGTGAT | TGAGTTGTATGTAGTCTCGTGGAT | Beta tubulin |

**Table S1**

**Primers used for qRT-PCR and RT-PCR.**


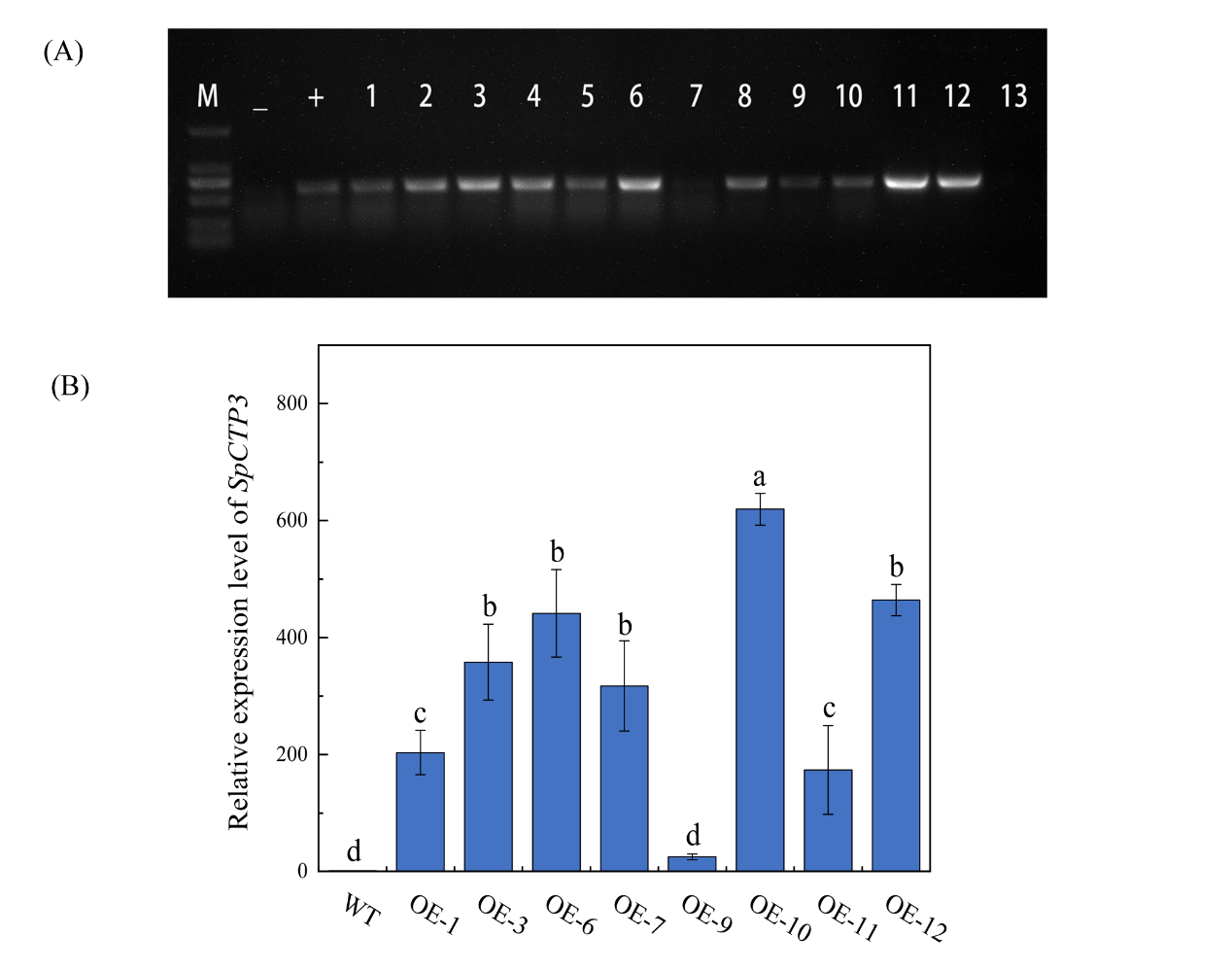


Fig.S1 Molecular identification of transgenic *SpCTP3* poplar plants. (A) PCR analysis of *SpCTP3* expression in transgenic plants using specific primers. pBI121-SpCTP3 plasmid was used as positive control, ddH_2_O was used as a negative control. - / +, control; 1-13, cDNA from transformants. M = DNA marker. (B) qRT-PCR analysis of transformants using quantified primers for *SpCTP3*. WT, untransformed control. Significantly different at the *p* < 0.05 level compared to WT
