# Supplementary figures and images for: A novel gene SpCTP3 from the hyperaccumulator Sedum plumbizincicola redistributes cadmium and increases its accumulation in transgenic Populus × canescens
Source: Front Plant Sci. 2023 Feb 8;14:1111789. doi: 10.3389/fpls.2023.1111789 (PMC9945123; doi:10.3389/fpls.2023.1111789)

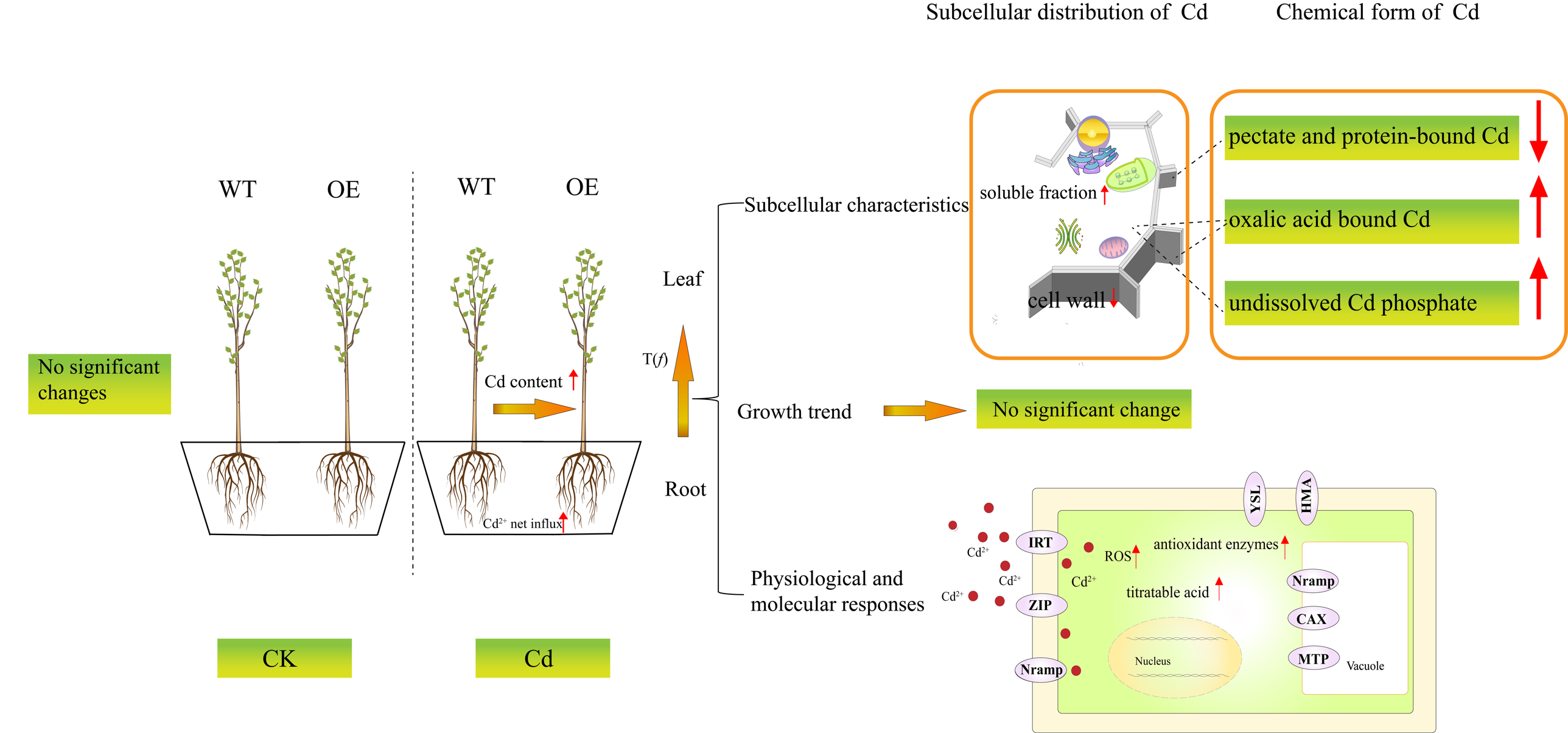

Supplement: Supplementary file 2 [file Image_1.tif]
